# Channels of Infection

**Published:** 1894-12-29

**Authors:** 


### Channels of Infection.

The study of tuberculosis is engaging some of our best men at the present time, both in medical, surgical, and veterinary practice, but the lessons taught by it apply also to other disease processes, and all who desire to become acquainted with the more recent phases of the question will gain much from a perusal of Professor Woodhead's admirable address before the North London Medico-Chirurgical Society. Amongst the various channels of infection we have direct inoculation of wounds in the skin and mucous membranes. Also inhalation by the respiratory passages, whereby the organism gains access to the lungs, there to undergo further growth and development. But there are other, and quite as important, channels of infection to which Professor Woodhead draws our attention. Around the entrance to the larynx and the upper part of the œsophagus is a ring of lymphoid tissue similar to that found in the walls of the intestine. Passing in and out of these patches of lymphoid tissue are leucocytes, or "wandering cells." These have the power to take up and absorb foreign substances, and so appear to be a means of protection against invasion by disease germs. The leucocytes appear able to attack and destroy the microbes and so prevent their further entrance into the system. But if the tissues and cells of the animal are not in a healthy condition the microbe may get the best of it, and then the leucocyte may succumb and form a source of nutriment to the microbe, which in this way gains access to the system. The patches of lymphoid tissue and the wandering cells, although normally acting as a protection to the animal, may thus under certain conditions fail in their purpose and become a way of entry for infection. We have then brought before us two very important routes of infection—by way of the pharyngeal tonsil on the one hand and the adenoid patches in the intestine on the other, both of which may lead, through the lymphatics, to infection of the glands at the root of the lungs, so that finally the lungs themselves become involved.

Whilst, therefore the lungs would appear to be the favourite habitat of the tubercular process, it is by no means certain that the pulmonary tissue is in every case primarily involved. During life the disease may show itself almost solely in the lungs, yet there is evidence to show that the starting point of the disease may not necessarily have been within the thoracic cavity. We see, then, that there is probably a great deal of truth in the idea of infection from food, and it may not be altogether a bad thing that the lay Press has taken up the question, for that may lead to the growth of a healthy public opinion, which shall demand in the near future a much more careful inspection of our milk and meat supplies than obtains at present.

In would thus appear that the many dangers attributed to the bacillus coli communis may have more foundation than some may imagine. Sevestre some years ago drew attention to the fact that improper feeding in children might lead to decomposition of the contents of the intestine, and result in fœtid diarrhœa and infective enteritis, from which a secondary general infection might occur, and in particular pulmonary congestion and broncho-pneumonia.

Micro-organisms can find a lodgment in the skin, but a local inflammation is necessary before they can effect an entrance into the circulation, but this, when it takes place, also occurs through the lymphatics. Acute rheumatism and osteo-myelitis have been attributed to infection through the tonsils, and according to Buschke the same channel of infection may be open to pyogenic microbes. From his researches it is probable indeed that the tonsils play a much more important rôle as the means of entrance of pus-producing organisms than the respiratory or alimentary tracts. Hence the care of the mouth and throat becomes of great importance, not only as a matter of local cleanliness, but also for the prevention of general disease.

